# Assessing Global Evidence on Cost-Effectiveness to Inform Development of Pakistan’s Essential Package of Health Services

**DOI:** 10.34172/ijhpm.2023.8005

**Published:** 2024-01-07

**Authors:** Maryam Huda, Nichola Kitson, Nuru Saadi, Saira Kanwal, Urooj Gul, Maarten Jansen, Sergio Torres-Rueda, Rob Baltussen, Ala Alwan, Sameen Siddiqi, Anna Vassall

**Affiliations:** ^1^Department of Community Health Sciences, Aga Khan University, Karachi, Pakistan; ^2^Department of Global Health and Development, London School of Hygiene and Tropical Medicine, London, UK; ^3^Health Planning Systems Strengthening and Information Analysis Unit (HPSIU), Ministry of National Health Services Regulations and Coordination, Islamabad, Pakistan; ^4^Department of Health Evidence, Radboud Institute of Health Sciences, Radboud University Medical Centre, Nijmegen, The Netherlands; ^5^Department of Health Evidence, Radboud University Medical Centre, Nijmegen, The Netherlands; ^6^DCP3 Country Translation Project, London School of Hygiene and Tropical Medicine, London, UK

**Keywords:** Cost-effectiveness, Transferability, Health Technology Assessment, Priority Setting

## Abstract

**Background:** Countries designing a health benefit package (HBP) to support progress towards universal health coverage (UHC) require robust cost-effectiveness evidence. This paper reports on Pakistan’s approach to assessing the applicability of global cost-effectiveness evidence to country context as part of a HBP design process.

**Methods:** A seven-step process was developed and implemented with Disease Control Priority 3 (DCP3) project partners to assess the applicability of global incremental cost-effectiveness ratios (ICERs) to Pakistan. First, the scope of the interventions to be assessed was defined and an independent, interdisciplinary team was formed. Second, the team familiarized itself with intervention descriptions. Third, the team identified studies from the Tufts Medical School Global Health Cost-Effectiveness Analysis (GH-CEA) registry. Fourth, the team applied specific knock-out criteria to match identified studies to local intervention descriptions. Matches were then cross-checked across reviewers and further selection was made where there were multiple ICER matches. Sixth, a quality scoring system was applied to ICER values. Finally, a database was created containing all the ICER results with a justification for each decision, which was made available to decision-makers during HBP deliberation.

**Results:** We found that less than 50% of the interventions in DCP3 could be supported with evidence of cost-effectiveness applicable to the country context. Out of 78 ICERs identified as applicable to Pakistan from the Tufts GH-CEA registry, only 20 ICERs were exact matches of the DCP3 Pakistan intervention descriptions and 58 were partial matches.

**Conclusion:** This paper presents the first attempt globally to use the main public GH-CEA database to estimate cost-effectiveness in the context of HBPs at a country level. This approach is a useful learning for all countries trying to develop essential packages informed by the global database on ICERs, and it will support the design of future evidence and further development of methods.

## Background

**Key Messages**

**Implications for policy makers**

- This paper examines how useful the global evidence base on cost-effectiveness was as a basis for defining the benefit package in Pakistan. As such its main messages relate to civil services and analysts who support policy-makers in evidence informed prioritization.
- When global evidence on cost-effectiveness was assessed from a low- and middle-income country (LMIC) perspective, we found that the global body of evidence could only be partially used, when considering whether the interventions from literature matched the delivery of the intervention in Pakistan.
- Our process provides transparency around the challenges associated with transferability of global evidence; clearly identifying when evidence is not applicable to country context and grading evidence quality, so that governments can make informed decisions.

**Implications for the public**

Our study encourages analysts and researchers to ensure transparency in estimates of cost-effectiveness used in priority setting exercises, rather than use of default values without clear indications of uncertainty related to cross setting transference. Our work also suggests that the global community producing cost-effectiveness analyses should clearly report interventions and comparators, report cost breakdowns to allow for local adjustment, and include scenario analyses to explore key contextual factors that may influence cost-effectiveness and may support the transference of results to multiple settings.

Universal health coverage (UHC) is based on the principle that all individuals and communities have access to essential, quality healthcare services with financial risk protection.^1^ Defining the health benefit package (HBP), or the essential package of health services (EPHS), is one of the first steps towards achieving UHC. An EPHS is a set of health services that can be feasibly financed and delivered to all citizens according to a country’s available resources.^2^ Defining an EPHS involves the selection and definition of decision criteria and assessing the performance of interventions against those criteria.^3^ A key criterion used to prioritize health interventions for inclusion in the EPHS and ensure efficient use of existing resources is cost-effectiveness.

 The Disease Control Priority 3 (DCP3) project provides a periodic review of the most up-to-date global evidence on cost-effectiveness of interventions to address the burden of disease in low-resource settings. DCP3 provides guidance on priority health interventions for UHC in the form of model UHC packages. The packages include an Essential UHC package (EUHC), comprising 218 interventions and the more limited High Priority Package, comprising a subset of 108 interventions, which could be adapted to reflect country-specific needs, health system capacities, financing structures, available resources, and other local circumstances. These interventions are recommended as a priority, based on an expert assessment of the evidence on cost-effectiveness globally.

 The full EUHC package, at 80% population coverage, is estimated to have a cost of 2016 US$ 79 per capita in low-income countries and US$ 130 per capita in low- and middle-income countries (LMIC),^4^ which exceeds current health expenditure in many settings. Therefore, further prioritization of the DCP3 model UHC package is required at the country level, tailored to local needs and by considering relevant evidence across several criteria, commonly including cost-effectiveness. Pakistan is one of the first countries to use the DCP3 model as the starting point for the design of a UHC EPHS and adapting it further using an evidence-informed deliberative process.

 Cost-effectiveness is a concept that is inherently context specific, and the cost-effectiveness of interventions will vary according to demographic, epidemiological and health system characteristics. If local cost-effectiveness evidence is unavailable, there are several approaches available for adapting or transferring estimates of cost-effectiveness across settings. One approach is to model cost-effectiveness ratios using local data. However, this can be time consuming and demands extensive capacity if many interventions need to be considered. An alternative is to apply frameworks typically used in the context of health technology assessment (HTA) to transfer cost-effectiveness results for specific new technologies across settings,^5^ one form of which is to extrapolate cost-effectiveness ratios from other settings, adjusting for country income groups (as was done by DCP3). HTA frameworks that adjust for a range of factors determining cost-effectiveness are often focused on single incremental interventions and require substantial data input, both from the context of the original estimate and the jurisdiction to which it is being applied. HBP design processes typically have timeframes of a year or less and can cover hundreds of interventions: it is thus unclear how feasible current transferability guidance may be for UHC EPHS design.

 This paper sets out the approach used by the Pakistan Ministry of National Health Services, Regulations and Coordination (MNHSR&C) to move beyond simple income-based extrapolation, and additionally assess the applicability of the global evidence base on cost-effectiveness to the country context, using a simplified transferability framework. The paper reflects on the appropriateness of the method used, and more broadly on the appropriateness of the existing global body of literature on cost-effectiveness for the purposes of EPHS design in LMICs.

## Methods

 The overall process of priority setting for the UHC EPHS in Pakistan was rooted in the approach outlined elsewhere,^3^ employing evidence-informed deliberation, whereby evidence is summarized and appraised in a systematic and transparent way by relevant stakeholders. Given the timeframe of the EPHS design process (six months to one year), and after review of the various models available, the MNHSR&C decided it was not possible to model cost-effectiveness for multiple interventions, using local data. It was therefore decided to use global estimates of cost-effectiveness summarized by DCP3 and transfer these to the country context by developing a novel approach that assesses the applicability of incremental cost-effectiveness ratios (ICERs) to Pakistan’s context.

 ICER is the most frequently used measure of cost-effectiveness calculated by dividing the difference in total costs between an intervention and comparator (incremental costs) by the difference in the chosen measure of the health outcome or effect (incremental effect) to provide an incremental ratio of ‘cost per unit of health effect.’^6^ ICERs can be a limited measure of cost-effectiveness in respect of EPHS design, where average cost-effectiveness ratios (ACERs) are sometimes used to examine the most efficient package assuming a null comparator. However, ACERs do not take into account both the shared costs and impact of different combinations of interventions, and hence in principle ICERs are more appropriate if looking at an expansion pathway to UHC. There is also very limited empirical evidence on ACERs, so in practice, and in the case of DCP3, ICERs are used as the measure of cost-effectiveness, despite the fact that they are highly unlikely to be estimated against the past and proposed combination of interventions being considered in EPHS processes.

 Our approach presumes that ICERs from other settings may be uncertain and biased, when applied to Pakistan; and that the overall process should ensure as much transparency about global evidence quality as was feasible within our timeframe. To this end, we developed an assessment process that did not transfer ICER values for Pakistan, but instead selected the most appropriate ICER and characterized the quality of the ICER in terms of relevance to the Pakistan context, as a novel means to facilitate the application of ICERs to Pakistan’s context, and in an easily interpretable manner for decision-makers unfamiliar with economic evaluation. Our aim was to facilitate critical stakeholder review of ICER estimates to arrive at consensus regarding interventions to include in the EPHS, using ICERs alongside expert judgement and appraisal. In this way our approach is in line with the approach of DCP3 globally, that combines expert judgement and literature on ICERs, accepting that the underlying literature base is incomplete and biased.

 The scope of our analysis took the DCP3 model EUHC package as a point of departure with 218 interventions divided into 4 clusters (reproductive, maternal, neonatal, child, and adolescent health and nutrition; infectious diseases; non-communicable diseases (NCDs); and health services access) across five delivery platform levels (community, primary healthcare [PHC], first-level hospital, referral hospital, and population level). These were further narrowed down by the MNHSR&C in an extensive consultation process to 170 interventions (including some that were splits of DCP3 interventions which were considered for assessment and appraisal for inclusion in the Pakistan EPHS) (Baltussen et al, unpublished data, 2023).

 To determine our general approach to assessing the applicability of ICERs to Pakistan, we reviewed tools and checklists from the HTA literature to try to aid the process and identify factors that would impact evidence quality.^7^ Most approaches involve an initial assessment to examine whether the study/evidence under consideration is a suitable candidate for transferability to a new setting. This initial assessment is often referred to as the ‘knock-out’ criteria, or the ‘minimal methodology standard’ and usually involves considering quality of the study, transparency of methods, level of reporting of methods and results, and applicability of the treatment comparators to the target country. A further assessment is then conducted based on other context specific factors using checklists, flowcharts and toolkits of criteria covering domains deemed to be important influences on transferability, for example the transferability of the health outcome data, the perspective, the study design, etc. Some approaches generate a quantitative score or index to measure transferability. The assessment criteria chosen by different authors varied widely in both content and extensiveness,^8^ for example Welte’s consists of three assessment criteria, in comparison to Boulanger’s transferability information checklist which is a 42-question tool.^9^

**Figure 1:**
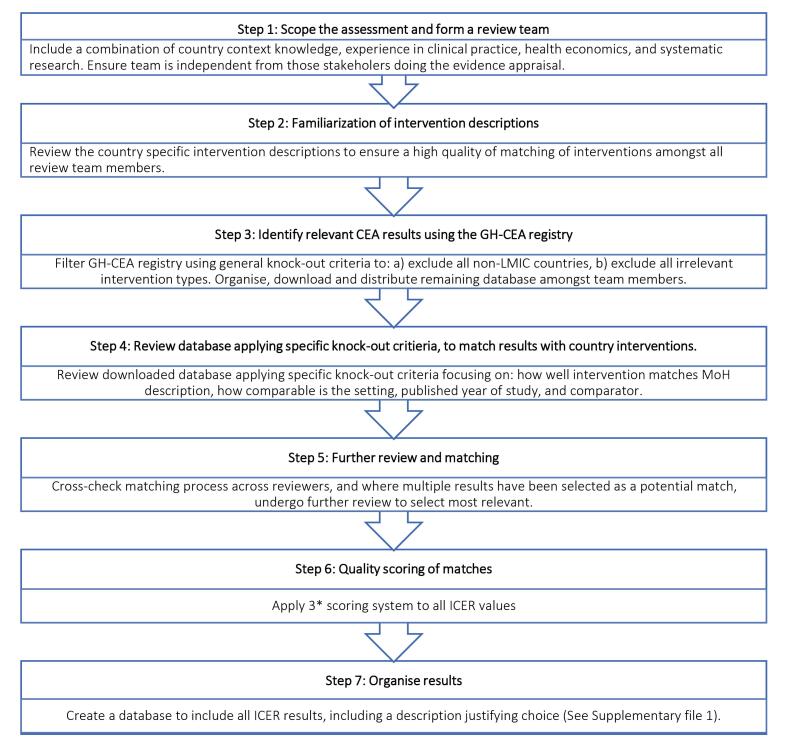
7-Step Process ‘Assessing ICERs for Health Benefit Package Design’ in Pakistan. Abbreviations: GH-CEA, Global Health Cost-Effectiveness Analysis; LMIC, low- and middle-income country; ICER, incremental cost-effectiveness ratio; MoH, Ministry of Health.

 After piloting several approaches to assess the feasibility within our time and resource constraints, it was decided that it was not feasible to adjust ICER values but instead to select the most appropriate ICER and characterize the quality of the ICER in terms of relevance to the Pakistan’s context. We developed and employed a 7-step process (Figure 1).

 The first step in our process was scoping the interventions to be assessed and forming an independent, interdisciplinary team. The review team consisted of five core members, and combined experience in health economics, research, and clinical practice. It consisted of two international DCP3 staff (health economist, systematic review expert), 1 local academic (clinical expert and health economist) and 2 MNHSR&C staff (clinical expert, statistician). In addition, the results were reviewed both by an additional senior international health economist, and the full DCP3 Secretariat at the MNHSR&C.

 The second step was for the team members to become familiar with the interventions being considered for the EPHS. The MNHSR&C prepared detailed intervention description sheets which described how the intervention will be implemented; at what platform, the population in need, the procedures, technologies, and medicines involved. These were reviewed by the core team members.

 Step three identifies studies to review as potential matches to the Pakistan-specific DCP3 interventions. Out of the total 170 interventions included in the assessment, we searched for ICERs for 166 interventions: 41 interventions at community level, 56 at PHC level, 49 interventions at the first-level hospitals and 20 interventions at referral hospital level. While not considered for inclusion in the EPHS at the district level, we also searched ICERs for 13 population-level interventions. No ICERs could be searched for four interventions because they were too broad in their definition. The four interventions considered too broad were: FLH57 – Prevention and relief of refractory suffering and acute pain related to surgery, serious injury or other serious, complex, or life-limiting health problems; FLH58 – First level hospital pathology services; HC67 – Expanded palliative care and pain control measures, including prevention and relief of all physical and psychological symptoms of suffering; and HC68 – Health center pathology services. A full list of interventions that were analyzed is contained in Table S1 of Supplementary file 1.

 We piloted several approaches to identify ICERs from the literature. The initial approach tried to use the systematic reviews prepared by DCP3 as the basis to identify the best country specific ICER estimate, and to update these reviews. However, the DCP3 database did not provide sufficient detail, nor were each of the searches (and study extraction methods) consistent across volumes. It was therefore not possible to re-do the systematic reviews of all DCP3 evidence within our time frame. We therefore used the Tufts Medical Center Global Health Cost-Effectiveness Analysis (GH-CEA) Registry,^15^ as the registry extracts all elements needed both in terms of ICER values and standard quality assessment tools, such as scoring against the Consolidated Health Economic Evaluation Reporting Standards (CHEERS) checklist.

 The GH-CEA registry extracts several outcomes from the global literature, including a “incremental cost-per-disability life year averted” metric, the same metric as used in DCP3, which enables comparability, but may bias searches towards newer technologies or studies on treatment, as disability-adjusted life years (DALYs) have been increasingly used over time. We have included the details of our search terms and process in Box S1 of Supplementary file 1. Once we had arrived at a set of studies to be evaluated, we downloaded the database into Excel. It was organized and distributed amongst team members for review. The downloaded file included the publication year, target population, study country, intervention description, comparator description, and incremental cost per DALY averted in current United States Dollar (USD). Table S2 of Supplementary file 2 contains the full list of the data extracted.

 In step four, each team member reviewed the studies for inclusion by applying specific knock-out criteria. We used Welte and colleagues’ general knock-out criteria, which is comprised of three factors: (1) the relevant technology (intervention) is not comparable to the one that shall be used in the decision country; (2) the comparator is not comparable to the one that is relevant to the decision country; and (3) the study does not possess an acceptable quality, according to a standard reporting checklist (CHEERS checklist).^10^ To assess the intervention for matching, the reviewers first reviewed the intervention description, extracted from the GH-CEA for each study to see how well they matched those provided by the MNHSR&C. Reviewers were asked to score whether there was an exact match, a partial or no match. An exact match refers to an intervention description from the GH-CEA results which matches the DCP3 Pakistan intervention description in terms of method, delivery, and technology used. A partial match refers to an intervention description from the GH-CEA results which only matches some of those elements. This process was completed by each reviewer blinded to other reviewers, and cross-checked by a second reviewer, followed by discussion. Those with exact or partial matches were paired with the relevant DCP3 intervention.

 Step five selected the most relevant ICER in case multiple studies survived the knock-out step. The pros and cons of each study in terms of matching to the Pakistani setting were discussed until one ICER value agreed to be the most relevant. Factors that favoured selection of the study included the most recent publication date, best intervention match, appropriate comparator, context specific factors such as service delivery level, or specific drug or vaccine used. Finally, a one-line justification was written to explain why a study was chosen.

 Step six scored the quality of the extracted ICERs by adding a simple three-star scoring system focusing on providing an indication of how applicable the ICER was to the Pakistani context. For three stars, the ICER result came from Pakistan, and was either a partial or exact match. To receive two stars, the ICER values came from a study from another LMIC setting and was either a partial or exact match. One star was given to interventions where a partial or exact match was not found.

 In step seven, we summarized the justification for why each ICER value was chosen for each intervention. This was made available to the stakeholders in long form and in simple evidence sheets, alongside evidence of costs and burden of disease. During the evidence deliberation sessions, the core team was available to answer any questions and was ready to provide access to full study texts if requested.

 Where we did not find any value, we used the default values from DCP3, with the lowest quality score. Finally, these values were entered into the Health Interventions Prioritization Tool, developed by University College London and The World Bank (See further details in Box S2 of Supplementary file 1). This tool adjusts ICERs by the attributable disease burden, where the impact generated cannot exceed total annual disease burden for the disease. Where this was done, we also gave the lowest quality to final ICER values presented to decision-makers.

## Results

**Figure 2:**
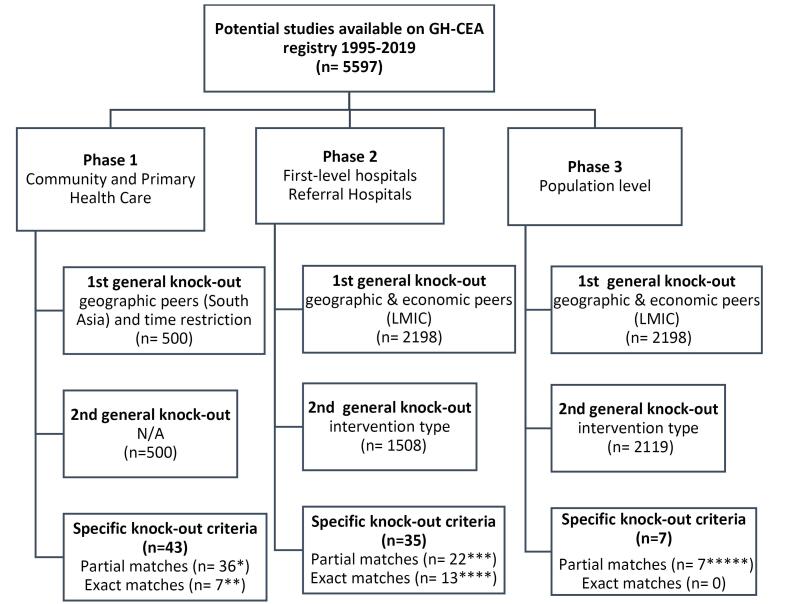
Search Process Using Tufts GH-CEA Registry. Abbreviations: GH-CEA, Global Health Cost-Effectiveness Analysis; LMIC, low- and middle-income country; N/A, not applicable.

Figure 2 presents the number of studies considered in each of the seven steps. The GH-CEA registry includes a total of 5597 studies from 1995-2019. After applying the general knock out criteria, we identified 500 studies for PHC (phase 1 of the EPHS) applying both the first and second knock-out criteria. During phase 2, for first level and referral level hospitals we identified 2198 and 1508 studies, after the first and second general knock out criteria, respectively. Finally, in phase 3 for population level interventions, we found 2198 and 2119 studies after applying the first and second general knock out criteria.

 Applying the specific knock-out criteria as part of step 4 in our process, we could only identify ICERS for 78 interventions that had relevant technology and quality. Of these, only 13 had a relevant comparator (See Table 1). Applying the quality scoring, almost 48% of these had a rating of two or three stars. The values of ICERs selected in step 5 can be found in Table S1 of Supplementary file 1.

**Table 1:** List of Interventions Which Had Evidence That Met Our Quality, Technology, and Comparator Criteria

**Interventions**	**Cluster**	**Platform**	**Quality**	**Technology**	**Comparator**
Antenatal and postpartum education on birth spacing	RMNCH	Community level	✓	✓	✓
Childhood vaccination series (diphtheria, pertussis, tetanus, polio, BCG, measles, hepatitis B, and HiB)	RMNCH	Community level	✓	✓	✓
Counselling of mothers on providing thermal care for pre-term new-borns (delayed bath and skin to skin contact)	RMNCH	Community level	✓	✓	✓
Promotion of breastfeeding or complementary feeding by community health workers	RMNCH	Community level	✓	✓	✓
Early detection and treatment of NTDs	Communicable diseases	Community level	✓	✓	✓
Acute severe malnutrition management	RMNCH	Community level	✓	✓	✓
Detection and treatment of childhood infections with danger signs (IMCI)	RMNCH	Health center	✓	✓	✓
Post-gender-based violence care, including counselling, provision of emergency contraception, and rape-response referral (medical and judicial)	RMNCH	Health center	✓	✓	✓
Partner notification and expedited treatment for common STIs including HIV	Communicable diseases	Health center	✓	✓	✓
Screening of HIV in all individuals with a diagnosis of active TB; if HIV infection is present, start (or refer for) ARV treatment and HIV care	Communicable diseases	Community level	✓	✓	✓
Screening for latent TB infection following a new diagnosis of HIV, followed by yearly screening among PLHIV at high risk of TB exposure; initiation of isoniazid preventive therapy among all individuals who screen positive but do not have evidence of active TB	Communicable diseases	Health center	✓	✓	✓
Provision of aspirin for all cases of suspected acute myocardial infarction	NCD and IPC	Health center	✓	✓	✓
Management of depression and anxiety disorders with psychological and generic antidepressants therapy	NCD and IPC	Health center	✓	✓	✓

Abbreviations: BCG, Bacillus Calmette–Guérin; HiB, hemophilic infection type b; STIs, sexually transmitted infections; TB, tuberculosis; PLHIV, people living with HIV; NCD, non-communicable disease; RMNCH, Reproductive, Maternal, Newborn and Child Health; IPC, injury prevention cluster; IMCI, integrated management of childhood illness; NTDs, neglected tropical diseases; ARV, antiretroviral.

 The proportion of interventions for which matches were found varied by platform (See Tables 2 and 3). At the community level, 18 interventions out of 41 were found from the Tufts GH-CEA registry. For the PHC level interventions, 25 out of the 56 ICERs were found from the Tufts GH-CEA registry. Out of these 43 GH-CEA studies, only 7 were exact matches of the DCP3 interventions and the study intervention, while 36 were partial matches. Thirteen studies were from Pakistan, 25 studies were from South Asia and 5 from other LMICs.

**Table 2:** Interventions’ Cluster, Search Results and Quality Scoring

**Platform/Level**	**No. of Interventions**	**Tufts GH-CEA Registry (78)**			**Final ICER Quality Scoring for All Interventions (166)**^a^		
		**Meeting Knockout Criteria 1 and 2**	**Exact/Partial Match**	**Pakistan/South Asia/LMIC Match**	*	**	***
Community	41	18	Exact = 5, Partial = 13	Pakistan = 6, South Asia = 10, LMIC = 2	23	12	6
Primary healthcare	56	25	Exact = 2, Partial = 23	Pakistan = 7, South Asia = 15, LMIC = 3	31	18	7
First level hospital	49	25	Exact = 8, Partial = 17	Pakistan = 0, South Asia = 1, LMIC = 24	24	25	-
Referral hospital	20	10	Exact = 5, Partial = 5	Pakistan = 0, South Asia = 0, LMIC = 10	10	10	-
Total	166	78	Exact = 20, Partial = 58	Pakistan = 13, South Asia = 26, LMIC = 39	88	65	13

Abbreviations: GH-CEA, Global Health Cost-Effectiveness Analysis; LMIC, low- and middle-income country; ICER, incremental cost-effectiveness ratio. ^a^This includes a * quality scoring for interventions if no ICER was available from the literature search and default DCP3 values were used.

**Table 3:** Quality Scoring of ICERs for Population Level Interventions

**Platform/Level**	**No. of interventions**	**Tufts GH-CEA registry**			**Final Quality Scoring for All ICERs**		
		**Meeting Knockout Criteria 1 and 2**	**Exact/Partial Match**	**Pakistan/South Asia/LMIC Match**	*	**	***
Population level	13	7	Exact = 0, Partial = 7	Pakistan = 2, South Asia = 2, LMIC = 3	2	9	2

Abbreviations: GH-CEA, Global Health Cost-Effectiveness Analysis; LMIC, low- and middle-income country; ICER, incremental cost-effectiveness ratio. This includes a * quality scoring for interventions if no ICER was available from the literature search and default DCP3 values were used.

 For the first level hospital interventions, 25 of the 49 ICERs were found from Tufts GH-CEA registry. Out of the 25 results, 8 were exact matches of the DCP3 interventions and the study intervention, while 17 were partial matches. One study was from South Asia and 24 were from other LMICs. Of the referral hospital interventions, 10 ICERs were from the Tufts GH-CEA registry, 5 were an exact match and 5 were a partial match. All 10 were from LMICs. Lastly, after a systematic search for ICERs for the 13 population level interventions, 7 were identified through the Tufts GH-CEA registry. Out of these 7, 2 were from Pakistan, 2 from South Asia and the remaining 3 from other LMICs. All 7 were partial matches.

**Figure 3:**
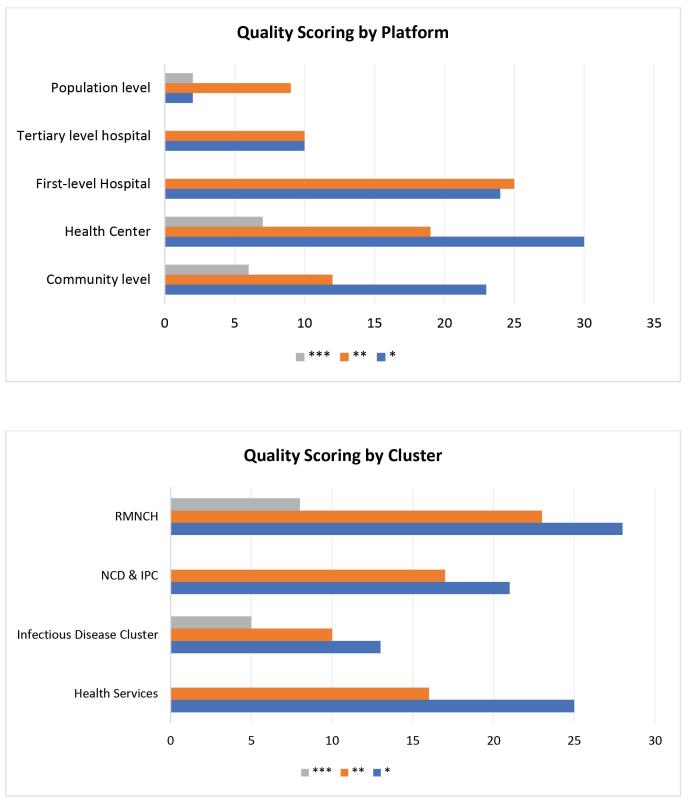
Quality Scoring by (a) Platform and (b) Cluster. Abbreviations: NCD, non-communicable disease; RMNCH, Reproductive, Maternal, Newborn and Child Health; IPC, injury prevention cluster.

 Quality scoring for each of the studies by platform and cluster is shown in Figure 3a and 3b, and more detail is provided in Table 3. Out of the total 166 interventions reviewed, 88 studies received one star, 65 studies received two stars and 13 studies received three stars. The remaining ICERs (default DCP3 values) were all scored one star.

## Discussion

 We have presented here a pragmatic but systematic approach to assess the applicability of the global cost-effectiveness evidence for use in HBP design processes. We found that, even when partial geographical and intervention matched ICERs were used, there was a dearth of context-relevant evidence on the cost-effectiveness of DCP3 interventions, with under 50% of the interventions receiving an ICER value that we could source from the incremental cost per DALY literature evidence base.

 The lack of sufficient economic evaluation evidence to inform priority setting in LMICs has been long noted.^11^ In simple terms, there are three approaches used to address this data gap: (*a*) modelling context specific estimates using local cost, effectiveness and epidemiological data, (*b*) extrapolation of the current evidence base across settings and (*c*) reviewing the literature, without adjustment, using expert judgement. At the global level, DCP3 attempted to facilitate those wishing to use the third approach locally, by conducting a global exercise to identify interventions where there is strong evidence of cost-effectiveness, to provide a long list of interventions for LMICs to include in benefit packages and to provide a broad estimation of cost-effectiveness by country income group. We found that a similar combination and expert review are likely to be required at the country level.

 Limiting our evidence review to studies found on the GH-CEA database restricted us to incremental cost per DALY averted studies. This limitation in part explains the gap in the evidence base between the scope of DCP3 global review of cost-effectiveness and our localized evidence of cost-effectiveness. DCP3 also has gaps, circumscribed by the overall economic literature, and does not include foundation non-disease specific interventions, such as routine symptom screening services. The exclusion of non-DALY studies biases towards more recent interventions. However, while evidence of cost-effectiveness, which does not estimate cost per DALY averted, may be appropriate when comparing interventions with the same outcomes, it is not appropriate for HBPs exercises, where a generic health outcome metric is required. Further work estimating the cost per DALY averted for economic evaluations that currently use other disease specific metrics is urgently required before any future DCP-type global exercises.

 We also found substantial differences in the numbers of studies available across interventions, which suggests that funding for economic evaluations in LMIC contexts may not be balanced from a health sector wide perspective. The interventions with the most substantial evidence base were typically those with potentially high commodity costs, such as Rotavirus vaccination. This publication bias is not surprising, as new (and high cost) interventions may be more likely to be subjected to HTA. Going forward, relying on a body of literature primarily geared to supporting incremental analyses may not best redress this evidence gap; and more investment is needed in economic evaluations targeting some of the gaps we found, once prior to DALY studies have been considered.

 In addition to empirical limitations, there are also theoretical concerns when transferring ICERs to support HBP prioritization. The main challenge is that ICERs are estimated as incremental to ‘a comparator’ that may not be appropriate for the context. We prioritized ICERs compared to a ‘do-nothing’ comparator, but we only found 13 studies that included this comparator; other ICERs intrinsically reflect the underlying status quo of health service delivery in the study country. An alternative approach is to generate an evidence base on ACERs compared to a null (no health service delivery), replicating building a health system up from nothing. However, ACERs cannot be validated empirically. It is unlikely that current health service delivery will be dismantled and rebuilt, and thus to some extent even the most radical reallocation of resources will also be in practice incremental, with ACERs being more a theoretical construct to help countries determine how far off the current system has shifted from optimal resource allocation.

 The above biases and limitations may be avoided if efforts are made to locally model ICERs. Infectious disease programs commonly conduct such exercises to inform resource allocation, and WHO CHOICE provides a framework for bringing some of those models with other analyses together to look at multiple interventions. WHO CHOICE was recently applied to benefit package revision in Ethiopia.^12^ In this case, it was able to cover around half the interventions. Ochalek and colleagues also combined both modelling and existing data when supporting the National Essential Health Package of Malawi.^13^ In our first feasibility assessment, modelling ICERs using WHO-CHOICE (World Health Organization CHOosing Interventions that are Cost-Effective) was considered in Pakistan as well. However, while WHO-CHOICE can produce results in short time frames, understanding the assumptions, epidemiologic models and costs driving those results sufficiently to judge their quality was not considered possible by the stakeholders within the available time frame. While the published evidence base is subject to the same complexity, the combination of peer review, quality assessment and finally stakeholder review (as part of the evidence-based deliberation) was chosen by the MNHSR&C for greater assurance of quality, transparency and stakeholder engagement, as important outcomes of EPHS prioritization processes, even if the empirical evidence may be of lower quality.

 Our experience of applying the DCP3 evidence in Pakistan highlights the challenges LMICs face when trying to use limited global evidence for UHC benefit package definition. While DCP3 reflects the consensus around a very broad package of essential services that can be adapted according to local needs and affordances, there remains a stark trade-off between satisfying the political and accountability imperatives to produce benefit packages rapidly and evidence quality. HBP design processes should thus not be seen as one-off exercises but allow for continual evidence review to refine packages over time, particularly in high cost, marginal interventions where quality evidence is not available in the short term.

 Future global efforts can support this effort by focusing on evidence review processes that incorporate both a general and transferability assessment of evidence from different regional perspectives, rather than simply adjusting ICERs or providing default values by country income level. Where ICERs are used from other settings, this should be done with full transparency of the uncertainty and biases in such approaches. Regional analyses and reviews, which include some degrees of contextual assessment, would assist countries, funders, and the research community focus effort towards the most important evidence gaps.

 At the country level, EPHS design processes need to be conducted using carefully designed evidence review processes, which allow time for stakeholders to understand and appraise the applicability of evidence to their context systematically and explicitly. Benefit package definition should be followed up with a process to monitor and evaluate the package as it is implemented, ideally producing local evidence on costs and cost-effectiveness, which can add to the global evidence base. Finally, further involvement and interaction between those assessing ICERs at the country level and those engaged in global reviews/modelling efforts are critical to develop a community of practice in this complex but important area of UHC policy.

## Ethical issues

 Ethical approvals were obtained from the London School of Hygiene and Tropical Medicine (21247) and Aga Khan University (2019-1992-5190); MoH clearance is being sought.

## Competing interests

 Authors declare that they have no competing interests.

## Funding

 This paper is part of a series of papers coordinated by the DCP3 Country Translation Project at the London School of Hygiene and Tropical Medicine, which is funded by the Bill & Melinda Gates Foundation [Grant OPP1201812]. The sponsor had no involvement in paper design; collection, analysis and interpretation of the data; and in the writing of the paper.

## Supplementary files


Supplementary file 1 contains Table S1, Box S1, and Box S2.

Supplementary file 2 contains Table S2.
