# Supplementary material for: Assessing Global Evidence on Cost-Effectiveness to Inform Development of Pakistan’s Essential Package of Health Services
Source: Int J Health Policy Manag. 2024 Jan 7;13:8005. doi: 10.34172/ijhpm.2023.8005 (PMC11607590; doi:10.34172/ijhpm.2023.8005)
Supplement: Supplementary file 1 — contains Table S1, Box S1, and Box S2. [file ijhpm-13-8005-s001.pdf]

Article title: Assessing global evidence on cost-effectiveness to inform development of Pakistan's Essential Package of Health Services

Journal name: International Journal of Health Policy and Management (IJHPM)

Authors' information: Maryam Huda[1], Nichola Kitson[2], Nuru Saadi[2], Saira Kanwal[3], Urooj Gul[3], Maarten Jansen[4], Sergio Torres-Rueda[2], Rob Baltussen[5], Ala Alwan[6], Sameen Siddiqi[1], Anna Vassall[2]*

[1]Department of Community Health Sciences, Aga Khan University, Karachi, Pakistan.

[2]Department of Global Health and Development, London School of Hygiene and Tropical Medicine, London, UK.

[3]Health Planning Systems Strengthening and Information Analysis Unit (HPSIU), Ministry of National Health Services Regulations and Coordination, Islamabad, Pakistan.

[4]Department of Health Evidence, Radboud Institute of Health Sciences, Radboud University Medical Centre, Nijmegen, The Netherlands.

[5]Department of Health Evidence, Radboud University Medical Centre, Nijmegen, The Netherlands.

[6]DCP3 Country Translation Project, London School of Hygiene and Tropical Medicine, London, UK.

***Correspondence to:** Anna Vassall; Email: Anna.Vassall@lshtm.ac.uk

Citation: Huda M, Kitson N, Saadi N, et al. Assessing global evidence on cost-effectiveness to inform development of Pakistan's essential package of health services. Int J Health Policy Manag. 2024;13:8005. doi:10.34172/ijhpm.2023.8005

Supplementary file 1

Table S1 – List of interventions from Figure 2

***Partial matches - community & PHC interventions**
1. Antenatal and postpartum education on birth spacing.
2. Education on handwashing and safe disposal of children's stool
3. Vitamin A and zinc for children
4. Childhood vaccination series (diphtheria, pertussis, tetanus, polio, BCG, measles, hepatitis B, HiB)
5. Vision pre-screening by teachers; vision tests and provision of ready-made glasses on-site by eye specialists/ trained medical officer.
6. Counselling of mothers on providing thermal care for pre-term new-born (delayed bath and skin to skin contact)
7. Community-based HIV testing and counseling (for example, mobile units and venue-based

	testing), with appropriate referral or linkage to care and immediate initiation of lifelong ART.
8.	Provision of condoms to key populations, including female sex workers, men have sex with men, people who inject drugs (IDU), transgender populations, and prisoners.
9.	Provision of Disposable syringes who inject drugs (IDU)
10.	Promotion of breastfeeding or complementary feeding by community health workers
11.	Early detection and treatment of NTDs
12.	Tetanus toxoid immunization among schoolchildren and among women attending antenatal care.
13.	Acute severe malnutrition management
14.	Early detection and treatment of neonatal pneumonia with oral antibiotics
15.	Detection and treatment of childhood infections with danger signs (IMCI)
16.	Psychological treatment for mood, anxiety, ADHD and disruptive behavior disorders in adolescents
17.	Post-gender-based violence care, including counselling, provision of emergency contraception, and rape-response referral (medical and judicial)
18.	Partner notification and expedited treatment for common STIs including HIV.
19.	Provider-initiated testing and counselling for HIV, STIs and hepatitis for all in contact with the health system in high- prevalence setting, including prenatal care with appropriate referral/ linkages to care including immediate ART initiation for those testing positives for HIV.
20.	IPT for TB contacts
21.	Diagnosis of TB and first-line treatment
22.	Screening of HIV in all individuals with a diagnosis of active TB; if HIV infection is present, start (or refer for) ARV treatment and HIV care.
23.	Screening for latent TB infection following a new diagnosis of HIV, followed by yearly screening among PLHIV at high risk of TB exposure; initiation of isoniazid preventive therapy among all individuals who screen positive but do not have evidence of active TB.
24.	Provision of insecticide treated nets to under five children and pregnant women attending health centers.
25.	Provision of aspirin for all cases of suspected acute myocardial infarction
26.	Opportunistic screening for hypertension for all adults and initiation of treatment among individuals with severe hypertension and/or multiple risk factors
27.	Tobacco cessation counselling and use of nicotine replacement therapy in certain circumstances.
28.	Provision of condoms, hormonal contraceptives including emergency contraceptives and IUDs.
29.	Provision of condoms, hormonal contraceptives including emergency contraceptives and IUDs.

30. Management of depression and anxiety disorders with psychological and generic antidepressants therapy
31. Screening and brief alcohol intervention
32. Targeted screening for congenital hearing loss in high-risk children, using optoacoustic testing otoscope.
33. TB ACF and linkage to care
****Exact matches- community & PHC interventions**
34. Pneumococcus vaccination
35. Rotavirus vaccination
36. School based HPV vaccination for girls
37. Provision of iron and folic acid supplementation to pregnant women, and provision of food or caloric supplementation to pregnant women in food-insecurity households (Community and PHC level)
38. Hepatitis B and C testing and referral
39. Low-dose inhaled corticosteroids and bronchodilators for asthma and for selected patients with COPD
*****Partial matches- first level and referral hospitals**
1. Management of severe acute malnutrition associated with serious infections.
2. Evaluation and management of fever in clinically unstable individuals using WHO IMAI guidelines, including empiric parenteral antimicrobials and antimalarial and resuscitative measures for septic shock.
3. Medical management of acute heart failure
4. Assisted vaginal delivery using vacuum extraction or forceps.
5. Craniotomy for trauma
6. Escharotomy or fasciotomy (Adults)
7. Hernia repair including emergency surgery for neonates and infants. (FLH and RH level)
8. Management of eclampsia with magnesium sulphate, including initial stabilization at health centers.
9. Management of septic arthritis
10. Placement of external fixator and use of traction for fractures.
11. Placement of external fixator and use of traction for fractures of Children.
12. Removal of gallbladder, including emergency surgery.
13. Repair of perforations (for example perforated peptic ulcer, typhoid ileal perforation)
14. Basic Skin grafting
15. Trauma laparotomy in Children (FLH and RH level)
16. Management of maternal sepsis, including early detection at health centers.

17. Compression therapy for amputations, burns, and vascular or lymphatic disorders.
18. Management of new-born complications, neonatal meningitis, and other very serious infections requiring continuous supportive care (such as IV fluids and oxygen)
19. Management of preterm labor with corticosteroids, including early detection at health centers.
20. Surgery for ectopic pregnancy (Also included in Surgery package of services)
21. Diabetes screening and care in pregnancy
22. For individuals testing positive for hepatitis B and C, assessment of treatment eligibility by trained providers followed by initiation and monitoring of ART when indicated.
23. Hepatitis B vaccination for high-risk populations, including healthcare workers, IDU, MSM, household contacts and partners with multiple sex partners (Also included in Cancer package of services)
24. Care for neonatal sepsis, pneumonia, meningitis
25. Full supportive care for preterm new-born
26. Elective surgical repair of common orthopedic injuries (for example meniscal and ligamentous tears) in individuals with severe functional limitation
27. Urgent, definitive surgical management of orthopedic injuries (for example open reduction and internal fixation)
28. Treatment of early-stage breast cancer with appropriate multimodal approaches (including generic chemotherapy) with curative intent for cases detected by clinical examination at health centers and first level hospitals.
29. Treatment of early-stage childhood cancers (such as Burkitt and Hodgkin lymphoma, acute lymphoblastic leukemia, retinoblastoma, and Wilms tumor) with curative intent in pediatric cancer units or hospitals
******Exact matches- first level and referral hospitals**
1. Early detection and treatment of early-stage cervical cancer (Also included in HIV and Cancer packages of services)
2. Management of acute coronary exacerbations of asthma and COPD using systemic steroids, inhaled beta-agonists and if indicated oral antibiotics and oxygen therapy.
3. In settings where sickle cell disease is a public health concern, universal newborn screening followed by standard prophylaxis against bacterial infections and malaria
4. Appendectomy
5. Fracture reduction
6. Hernia repair including emergency surgery.
7. Hysterectomy for uterine rupture or intractable postpartum hemorrhage
8. Trauma laparotomy
9. Among all individuals who are known to be HIV+, immediate ART initiation with regular

monitoring of viral load for (Also included in HIV package of services)
10. Cataract extraction and insertion of intraocular lens
11. Surgery for Trachomatous Trichiasis
12. Management of acute ventilator failure due to acute exacerbations of asthma and COPD
13. Retinopathy screening via telemedicine, followed by treatment using laser photocoagulation.
14. Treatment of early-stage colorectal cancer with appropriate multimodal approaches (including generic chemotherapy) with curative intent for cases detected by clinical examination at health centers and first level hospitals
*****Partial matches -Population**
1. Mass media messages concerning sexual and reproductive health and mental health for adolescents (Also included in HIV and Mental health packages of services)
2. Develop plans and legal authority for curtaining interactions between infected persons and un-infected population and implement and evaluate infection control measures in health facilities.
3. Mass media messages concerning awareness on handwashing and health effects of household air pollution.
4. Mass media messages concerning healthy eating or physical activity (Also included in CVD and Musculoskeletal packages of services)
5. Mass media messages concerning use of tobacco and alcohol (Also included in CVD and Musculoskeletal packages of services)
6. Mass media encouraging use of condoms, voluntary medical male circumcision and STI testing.
7. Sustained integrated vector management for effective control of visceral Leishmaniasis, dengue, chikungunya, CCHF and other vector borne NTDs

Box S1 - HIPtool

The HIPtool is a presentational aid, in that it does not estimate costs, impact or cost-effectiveness from resource use, price and epidemiological inputs directly. Instead, it is uses pre-specified costs, coverage, ICERs and burden of disease data to explore the total health sector cost and impact of different scenarios of interventions included in the HBP. The HIPtool was selected for two reasons 1) it is designed around the DCP3 intervention structure and 2) its data requirements were considered feasible by the MoNHSR&C.

Analyses to support HBP prioritisation typically use ACERs and may also us a pathway of ICERs that are dependent on the ordering of interventions. The HIPtool estimates neither, as ACERs are commonly not available in the literature and calculating optimal choice using ICERs requires a substantial dataset on the interaction of disease burden and economies of both scale and scope.The HIPtool comes pre-populated with ICER values for the 218 EUHC interventions. For 122 of these interventions the HIPtool uses specific ICER values from Annex 7A* – supplementary material for Chapter 7, Volume 9 of DCP3 - which lists the cost per DALY averted arrived at after a review of studies published between 2001-2016 globally. For the remaining 96 interventions, the HIPtool pre-populates based on the assumption that the provided EUHC interventions are cost-effective and sets the ICER value as equal to cost-effectiveness at the margin of current health sector spend as estimated by York.

* Available at: http://dcp-3.org/sites/default/files/chapters/Annex%207A.%20Details%20of%20Interventions%20in%20Figs.pdf

Box S2 - Details of search terms and processes in TUFTS

Step 1: A total of **5,597** *of* ***quality*** potential interventions were available on GH-CEA registry *(can assume level which improves transferability).*

Step 2: After first ‘knock-out’ based on **geographic/economic** peers, **2,198** searches were left *(closer contextually which improves transferability)*. This was done by excluding non-LMIC (according to WB classification)

Step 3: After the second ‘knock-out’ based on **relevancy of intervention, 1,508** searches remained *(improves transferability)*. This was achieved by **excluding**: Environmental Augmentation; Environmental Remediation; Immunization; Legislation; Psychosocial and **including**: Care delivery; Diagnostic; Health Education and Behavior; Maternal and neonatal care; Medical device; Medical procedure; Nutrition; Others; Pharmaceuticals; Reproductive; Screening; Surgical.

This was followed by 300 searches reviewed per team member.

- ‘knock-out’ based on **intervention match**
  - Exact, partial, no match
- ‘knock-out’ based on **geography (**Pakistan; Bangladesh, India & Bhutan; rest of LMIC)
