# Supplementary material for: Assessing Global Evidence on Cost-Effectiveness to Inform Development of Pakistan’s Essential Package of Health Services
Source: Int J Health Policy Manag. 2024 Jan 7;13:8005. doi: 10.34172/ijhpm.2023.8005 (PMC11607590; doi:10.34172/ijhpm.2023.8005)
Supplement: Supplementary file 2 — contains Table S2. [file ijhpm-13-8005-s002.pdf]

Article title: Assessing global evidence on cost-effectiveness to inform development of Pakistan's Essential Package of Health Services

Journal name: International Journal of Health Policy and Management (IJHPM)

Authors' information: Maryam Huda[1], Nichola Kitson[2], Nuru Saadi[2], Saira Kanwal[3], Urooj Gul[3], Maarten Jansen[4], Sergio Torres-Rueda[2], Rob Baltussen[5], Ala Alwan[6], Sameen Siddiqi[1], Anna Vassall[2]*

[1]Department of Community Health Sciences, Aga Khan University, Karachi, Pakistan.

[2]Department of Global Health and Development, London School of Hygiene and Tropical Medicine, London, UK.

[3]Health Planning Systems Strengthening and Information Analysis Unit (HPSIU), Ministry of National Health Services Regulations and Coordination, Islamabad, Pakistan.

[4]Department of Health Evidence, Radboud Institute of Health Sciences, Radboud University Medical Centre, Nijmegen, The Netherlands.

[5]Department of Health Evidence, Radboud University Medical Centre, Nijmegen, The Netherlands.

[6]DCP3 Country Translation Project, London School of Hygiene and Tropical Medicine, London, UK.

***Correspondence to:** Anna Vassall; Email: Anna.Vassall@lshtm.ac.uk

Citation: Huda M, Kitson N, Saadi N, et al. Assessing global evidence on cost-effectiveness to inform development of Pakistan's essential package of health services. Int J Health Policy Manag. 2024;13:8005. doi:10.34172/ijhpm.2023.8005

Supplementary file 2

Table S2 – Detailed list of final ICERs

Intervention name	Geography: Pakistan/ South Asia/ Other LMIC	Exact OR Partial Match	Quality Scoring (1, 2 OR 3 stars)	FINAL ICER Value	ICER Source
Antenatal and postpartum education on birth spacing	Pakistan	Partial	***	40	Tufts GH-CEA registry
Education on handwashing and safe disposal of children's stool	South Asia	Partial	**	24	Tufts GH-CEA registry
Pneumococcus vaccination	South Asia	Exact	**	524	Tufts GH-CEA registry, with adjustment
Rotavirus vaccination	Pakistan	Exact	***	6620	Tufts GH-CEA registry, with adjustment

Vitamin A and zinc for children	South Asia	Partial	**	950	Tufts GH-CEA registry, with adjustment
Childhood vaccination series (diphtheria, pertussis, tetanus, polio, BCG, measles, hepatitis B, HiB)	LMIC	Partial	**	85	Tufts GH-CEA registry
Indoor residual spraying		Default value	*	151	DCP3 Annex 7a, with adjustment
Education of schoolchildren on oral health		Default value	*	757	DCP3 - No evidence, middle calculated York method
Vision pre-screening by teachers; vision tests and provision of ready-made glasses on-site by eye specialists/ trained medical officer	LMIC	Partial	**	160	Tufts GH-CEA registry
Counselling of mothers on providing thermal care for pre-term new-borns (delayed bath and skin to skin contact)	South Asia	Partial	**	38	Tufts GH-CEA registry
School based HPV vaccination for girls	Pakistan	Exact	***	2095	Tufts GH-CEA registry, with adjustment
Mass drug administration (NTDs)		Default value	*	3670	DCP3 Annex 7a, with adjustment
Adolescent-friendly services for STIs		Default value	*	757	DCP3 - No evidence, middle calculated York method
Life skills training in schools		Default value	*	757	DCP3 - No evidence, middle calculated York method
Provision of iron and folic acid supplementation to pregnant women, and provision of food or caloric supplementation to pregnant women in food-insecurity households	South Asia	Exact	**	186	Tufts GH-CEA registry
Provision of iron and folic acid supplementation to pregnant women, and provision of food or caloric supplementation to pregnant women in food-insecurity households	South Asia	Exact	**	186	Tufts GH-CEA registry

Community-based HIV testing and counseling (for example, mobile units and venue-based testing), with appropriate referral or linkage to care and immediate initiation of lifelong ART	Pakistan	Partial	***	200	Tufts GH-CEA registry
Provision of condoms to key populations, including female sex workers, men have sex with men, people who inject drugs (IDU), transgender populations, and prisoners	Pakistan	Partial	***	200	Tufts GH-CEA registry
Provision of Disposable syringes who inject drugs (IDU)	Pakistan	Partial	***	200	Tufts GH-CEA registry
TB contact tracing		Default value	*	757	DCP3 - No evidence, middle calculated York method
Test of malaria suspects and treatment		Default value	*	757	DCP3 - No evidence, middle calculated York method
Environmental management for malaria		Default value	*	222	DCP3 Annex 7a
Low-risk labor and delivery		Default value	*	1	DCP3 Annex 7a
Basic neonatal resuscitation following delivery		Default value	*	1	DCP3 Annex 7a
Management of labour and delivery in low risk women by skilled attendants		Default value	*	3	DCP3 Annex 7a, with adjustment
Basic neonatal resuscitation following delivery		Default value	*	1	DCP3 Annex 7a
Promotion of breastfeeding or complementary feeding by community health workers	South Asia	Partial	**	38	Tufts GH-CEA registry
Mass drug administration (malaria)		Default value	*	121	DCP3 Annex 7a, with adjustment
Early detection and treatment of NTDs	South Asia	Partial	**	740	Tufts GH-CEA registry, with adjustment
Identify and refer patients with high risk		Default value	*	757	DCP3 - No evidence, middle calculated York method

Guidance during infectious outbreak		Default value	*	757	DCP3 - No evidence, middle calculated York method
Exercise-based pulmonary rehabilitation		Default value	*	757	DCP3 - No evidence, middle calculated York method
Self-managed treatment of migraine		Default value	*	757	DCP3 - No evidence, middle calculated York method
Tetanus toxoid immunization among schoolchildren and among women attending antenatal care	South Asia	Partial	**	226	Tufts GH-CEA registry, with adjustment
Parent training of high-risk families, including nurse home visitation for child maltreatment		Default value	*	757	DCP3 - No evidence, middle calculated York method
WASH behavior change interventions		Default value	*	757	DCP3 - No evidence, middle calculated York method
ECD rehabilitation interventions		Default value	*	757	DCP3 - No evidence, middle calculated York method
Early childhood development rehabilitation interventions including motor, sensory and language stimulation		Default value	*	757	DCP3 - No evidence, middle calculated York method
Interventions for wheelchair users		Default value	*	757	DCP3 - No evidence, middle calculated York method
Acute severe malnutrition management	South Asia	Partial	**	1030	Tufts GH-CEA registry, with adjustment
Integrated community case management		Default value	*	757	DCP3 - No evidence, middle calculated York method

Care for fetal growth restriction		Default value	*	757	DCP3 - No evidence, middle calculated York method
Surgical termination of pregnancy		Default value	*	757	DCP3 - No evidence, middle calculated York method
Full supportive care for severe childhood infections with danger signs		Default value	*	757	DCP3 - No evidence, middle calculated York method
Management of severe acute malnutrition associated with serious infections	South Asia	Partial	**	860	Tufts GH-CEA registry, with adjustment
Early detection and treatment of early stage cervical cancer (Also included in HIV and Cancer packages of services)	LMIC	Exact	**	390	Tufts GH-CEA registry
Insertion and removal of long-lasting contraceptives(IUDs and Implants)(Also included in Surgery package of services)		Default value	*	757	DCP3 - No evidence, middle calculated York method
Tubal ligation		Default value	*	1150	DCP3 - No evidence, middle calculated York method , with adjustment
Vasectomy		Default value	*	757	DCP3 - No evidence, middle calculated York method
Referral of cases of treatment failure for drug susceptibility testing; enrolment of those with MDR-TB for treatment per WHO guidelines (either short or long term regimen)		Default value	*	220	DCP3 Annex 7a
Evaluation and management of fever in clinically unstable individuals using WHO IMAI guidelines, including empiric parenteral antimicrobials and antimalarial and resuscitative measures for septic shock	LMIC	Partial	**	1	Tufts GH-CEA registry

Induction of labor post-term		Default value	*	757	DCP3 - No evidence, middle calculated York method
Management of acute coronary syndromes with aspirin, unfractionated heparin and generic thrombolytic (when indicated)		Default value	*	3215	DCP3 Annex 7a
Management of acute ~~coronary~~ exacerbations of asthma and COPD using systemic steroids, inhaled beta-agonists and if indicated oral antibiotics and oxygen therapy	LMIC	Exact	**	11000	Tufts GH-CEA registry
Medical management of acute heart failure	LMIC	partial	**	195	Tufts GH-CEA registry, with adjustment
Bowel obstruction management		Default value	*	52	DCP3 Annex 7a, with adjustment
Secondary prevention of osteoporosis		Default value	*	1530	DCP3 - No evidence, middle calculated York method , with adjustment
Therapy for moderate to severe arthritis		Default value	*	420	DCP3 Annex 7a, with adjustment
In settings where sickle cell disease is a public health concern, universal new born screening followed by standard prophylaxis against bacterial infections and malaria	LMIC	Exact	**	200	Tufts GH-CEA registry
Identification of genetic disorders		Default value	*	757	DCP3 - No evidence, middle calculated York method
Jaundice management with phototherapy		Default value	*	757	DCP3 - No evidence, middle calculated York method
Management of intoxication / poisoning syndromes using widely available agents e.g. charcoal, naloxone, bicarbonate, antivenin		Default value	*	757	DCP3 - No evidence, middle calculated York method

Appendectomy	LMIC	Exact	**	1370	Tufts GH-CEA registry, with adjustment
Assisted vaginal delivery using vacuum extraction or forceps	LMIC	Partial	**	890	Tufts GH-CEA registry, with adjustment
Craniotomy for trauma	LMIC	Partial	**	200	Tufts GH-CEA registry
Colostomy		Default value	*	60	DCP3 Annex 7a
Escharotomy or fasciotomy (Adults)	LMIC	Partial	**	193	Tufts GH-CEA registry, with adjustment
Fracture reduction	LMIC	Exact	**	110	Tufts GH-CEA registry
Hernia repair including emergency surgery	LMIC	Exact	**	760	Tufts GH-CEA registry, with adjustment
Hernia repair including emergency surgery for neonates and infants	LMIC	Partial	**	7	Tufts GH-CEA registry
Hysterectomy for uterine rupture or intractable postpartum haemorrhage	LMIC	Exact	**	30	Tufts GH-CEA registry, with adjustment
Irrigation and debridement of open fractures		Default value	*	287	DCP3 Annex 7a
Management of eclampsia with magnesium sulphate, including initial stabilization at health centres	LMIC	Partial	**	103	Tufts GH-CEA registry, with adjustment
Osteomyelitis management		Default value	*	559	DCP3 Annex 7a
Management of septic arthritis	LMIC	Partial	**	370	Tufts GH-CEA registry
Placement of external fixator and use of traction for fractures	LMIC	Partial	**	370	Tufts GH-CEA registry
Placement of external fixator and use of traction for fractures of Children	LMIC	Partial	**	370	Tufts GH-CEA registry
Relief of urinary obstruction		Default value	*	510	DCP3 Annex 7a, with adjustment
Removal of gallbladder, including emergency surgery	LMIC	Partial	**	240	Tufts GH-CEA registry, with adjustment
Repair of perforations (for example perforated peptic ulcer, typhoid ileal perforation)	LMIC	Partial	**	12.25	Tufts GH-CEA registry, with adjustment

Resuscitation with advanced measures		Default value	*	757	DCP3 - No evidence, middle calculated York method
Basic Skin grafting	LMIC	Partial	**	560	Tufts GH-CEA registry
Trauma laparotomy	LMIC	Exact	**	14	Tufts GH-CEA registry
Trauma laparotomy in Children	LMIC	Partial	**	14	Tufts GH-CEA registry
Trauma related amputations		Default value	*	23	DCP3 Annex 7a
Management of maternal sepsis, including early detection at health centres	LMIC	Partial	**	940	Tufts GH-CEA registry, with adjustment
Tube thoracostomy		Default value	*	757	DCP3 - No evidence, middle calculated York method
Compression therapy for amputations, burns, and vascular or lymphatic disorders	LMIC	Partial	**	0	Tufts GH-CEA registry
Acute management of swallowing dysfunction		Default value	*	757	DCP3 - No evidence, middle calculated York method
Management of new-born complications, neonatal meningitis, and other very serious infections requiring continuous supportive care (such as IV fluids and oxygen)	LMIC	Partial	**	26	Tufts GH-CEA registry, with adjustment
Management of preterm labour with corticosteroids, including early detection at health centres	LMIC	Partial	**	25000	Tufts GH-CEA registry
Management of labour and delivery in high-risk women, including operative delivery (CEmONC) (Also included in Surgery package of services)		Default value	*	2592	DCP3 Annex 7a
Surgery for ectopic pregnancy (Also included in Surgery package of services)	LMIC	Partial	**	58	Tufts GH-CEA registry, with adjustment
Early detection and treatment of neonatal pneumonia with oral antibiotics	Pakistan	Partial	***	29	Tufts GH-CEA registry
Diabetes screening and care in pregnancy	South Asia	Partial	**	1800	Tufts GH-CEA registry

BEmNOC		Default value	*	187	DCP3 Annex 7a
Detection and treatment of childhood infections with danger signs (IMCI)	Pakistan	Partial	***	16	Tufts GH-CEA registry
Among all individuals who are known to be HIV+, immediate ART initiation with regular monitoring of viral load for (Also included in HIV package of services)	LMIC	Exact	**	2700	Tufts GH-CEA registry
Psychological treatment for mood, anxiety, ADHD and disruptive behaviour disorders in adolescents	South Asia	Partial	**	3375	Tufts GH-CEA registry
Post-gender-based violence care, including counselling, provision of emergency contraception, and rape-response referral (medical and judicial)	LMIC	Partial	**	844.03	Tufts/ICD-10
Management of sexual and reproductive tract infections		Default value	*	128	DCP3 Annex 7a, with adjustment
For individuals testing positive for hepatitis B and C, assessment of treatment eligibility by trained providers followed by initiation and monitoring of ART when indicated	South Asia	Partial	**	425	Tufts GH-CEA registry, with adjustment
Miscarriage and abortions management		Default value	*	757	DCP3 - No evidence, middle calculated York method
Hepatitis B and C testing and referral		Exact	*	353	Paper from peter
Partner notification and expedited treatment for common STIs including HIV	South Asia	Partial	**	109	Tufts GH-CEA registry
Provider-initiated testing and counselling for HIV, STIs and hepatitis for all in contact with the health system in high-prevalence setting, including prenatal care with appropriate referral/ linkages to care including immediate ART initiation for those testing positives for HIV	Pakistan	Partial	***	300	Tufts GH-CEA registry

Hepatitis B vaccination for high risk populations, including healthcare workers, IDU, MSM, household contacts and partners with multiple sex partners (Also included in Cancer package of services)	LMIC	Partial	**	270	Tufts GH-CEA registry
Medical male circumcision		Default value	*	757	DCP3 - No evidence, middle calculated York method
IPT for TB contacts	South Asia	Partial	**	190	Tufts GH-CEA registry
Diagnosis of TB and first-line treatment	LMIC	Partial	**	17	TB Paper, with adjustment
Screening of HIV in all individuals with a diagnosis of active TB; if HIV infection is present, start (or refer for) ARV treatment and HIV care	South Asia	Partial	**	3	Tufts/ICD-10 - negative value, adjustment made
Screening for latent TB infection following a new diagnosis of HIV, followed by yearly screening among PLHIV at high risk of TB exposure; initiation of isoniazid preventive therapy among all individuals who screen positive but do not have evidence of active TB	South Asia	Partial	**	1	Tufts/ICD-10 - negative value, adjustment made
Preterm premature rupture management		Default value	*	2128	DCP3 Annex 7a
Fever management for clinically stable		Default value	*	757	DCP3 - No evidence, middle calculated York method
Provision of insecticide treated nets to under five children and pregnant women attending health centres	Pakistan	Partial	***	200	Tufts GH-CEA registry
Identify and refer for progressive illness		Default value	*	757	DCP3 - No evidence, middle calculated York method
Therapy for CVD risk factors		Default value	*	757	DCP3 - No evidence, middle calculated York method

Low-dose inhaled corticosteroids and bronchodilators for asthma and for selected patients with COPD	South Asia	Exact	**	17626.34	Tufts/ICD-10
Provision of aspirin for all cases of suspected acute myocardial infarction	South Asia	Partial	**	310	Tufts GH-CEA registry
Screening for kidney disease		Default value	*	6116	DCP3 Annex 7a
Management of albuminuric kidney disease with ACEi		Default value	*	6116	DCP3 Annex 7a
Secondary prophylaxis for rheumatic fever		Default value	*	757	DCP3 - No evidence, middle calculated York method
Treatment of acute pharyngitis		Default value	*	15	DCP3 Annex 7a
Opportunistic screening for hypertension for all adults and initiation of treatment among individuals with severe hypertension and/or multiple risk factors	South Asia	Partial	**	400	Tufts GH-CEA registry
Tobacco cessation counselling and use of nicotine replacement therapy in certain circumstances	South Asia	Partial	**	210	Tufts GH-CEA registry
Support for caregivers of dementia patients		Default value	*	757	DCP3 - No evidence, middle calculated York method
Bipolar disorder management		Default value	*	1905	DCP3 Annex 7a
Provision of condoms, hormonal contraceptives including emergency contraceptives and IUDs	Pakistan	Partial	***	200	Tufts GH-CEA registry
Provision of condoms, hormonal contraceptives including emergency contraceptives and IUDs	Pakistan	Partial	***	200	Tufts GH-CEA registry
Management of depression and anxiety disorders with psychological and generic antidepressants therapy	South Asia	Partial	**	4591	Tufts GH-CEA registry
Screening and brief alcohol intervention	South Asia	Partial	**	4591	Tufts GH-CEA registry
Primary prevention of osteoporosis		Default value	*	757	DCP3 - No evidence, middle calculated York method

Targeted screening for congenital hearing loss in high-risk children, using optoacoustic testing otoscope	South Asia	Partial	**	1300	Tufts GH-CEA registry
Dental extraction		Default value	*	700	DCP3 - No evidence, middle calculated York method , with adjustment
Dental extraction (FLH)		Default value	*	757	DCP3 - No evidence, middle calculated York method
Drainage of dental abscess		Default value	*	1780	DCP3 - No evidence, middle calculated York method , with adjustment
Drainage of dental abscess (FLH)		Default value	*	3100	DCP3 - No evidence, middle calculated York method , with adjustment
Drainage of superficial abscess		Default value	*	1501	DCP3 - No evidence, middle calculated York method , with adjustment
Counseling on kangaroo care for newborns		Default value	*	301	DCP3 Annex 7a
Counselling of mothers on providing kangaroo care of new-borns		Default value	*	301	DCP3 Annex 7a
Care for neonatal sepsis, pneumonia, meningitis	Pakistan	Partial	***	75	Tufts GH-CEA registry
Non-displaced fractures management		Default value	*	757	DCP3 - No evidence, middle calculated York method
Resuscitation with basic measures		Default value	*	757	DCP3 - No evidence, middle calculated York method

Suturing laceration		Default value	*	757	DCP3 - No evidence, middle calculated York method
Treatment of caries		Default value	*	4650	DCP3 - No evidence, middle calculated York method , with adjustment
Treatment of caries (FLH)		Default value	*	4550	DCP3 - No evidence, middle calculated York method , with adjustment
Basic management of MNIs and disorders		Default value	*	7583	DCP3 Annex 7a
Psychosocial support and counseling		Default value	*	757	DCP3 - No evidence, middle calculated York method
Pharmacological termination of pregnancy		Default value	*	59	DCP3 Annex 7a, with adjustment
Care for hypertensive disorders in pregnancy		Default value	*	92504	DCP3 Annex 7a
Screening and management of hypertensive disorders in pregnancy		Default value	*	92504	DCP3 Annex 7a
TB ACF and linkage to care	South Asia	Partial	**	2500	Tufts GH-CEA registry
Full supportive care for preterm new-borns	LMIC	Partial	**	58	Tufts GH-CEA registry
Elective surgical repair of common orthopaedic injuries (for example meniscal and ligamentous tears) in individuals with severe functional limitation	LMIC	Partial	**	110	Tufts GH-CEA registry
Urgent, definitive surgical management of orthopaedic injuries (for example open reduction and internal fixation)	LMIC	Partial	**	370	Tufts GH-CEA registry
Repair of cleft lip and cleft palate		Default value	*	7900	DCP3 Annex 7a, with adjustment

Repair of club foot		Default value	*	111	DCP3 Annex 7a, with adjustment
Cataract extraction and insertion of intraocular lens	LMIC	Exact	**	1600	Tufts GH-CEA registry, with adjustment
Repair of anorectal malformations		Default value	*	757	DCP3 - No evidence, middle calculated York method
Repair of obstetric fistula		Default value	*	757	DCP3 - No evidence, middle calculated York method
Insertion of shunt for hydrocephalus		Default value	*	158	DCP3 Annex 7a
Surgery for Trachomatous Trichiasis	LMIC	Exact	**	370	Tufts GH-CEA registry
Referral level hospital pathology services		Default value	*	757	DCP3 - No evidence, middle calculated York method
Specialized TB services, including management of MDR- and XDR-TB treatment failure and surgery for TB		Default value	*	757	DCP3 - No evidence, middle calculated York method
Speciality pathology services		Default value	*	757	DCP3 - No evidence, middle calculated York method
Management of refractory febrile illness including etiologic diagnosis at reference microbial laboratory		Default value	*	757	DCP3 - No evidence, middle calculated York method
Management of acute ventilator failure due to acute exacerbations of asthma and COPD	LMIC	Exact	**	11000	Tufts GH-CEA registry
Retinopathy screening via telemedicine, followed by treatment using laser photocoagulation	LMIC	Exact	**	220	Tufts GH-CEA registry

Use of percutaneous coronary intervention for acute myocardial infarction where resources permits		Default value	*	673	DCP3 Annex 7a
Treatment of early stage breast cancer with appropriate multimodal approaches (including generic chemotherapy) with curative intent for cases detected by clinical examination~~ at health centres and first level hospitals~~	LMIC	Partial	**	6500	Tufts GH-CEA registry
Treatment of early stage colorectal cancer with appropriate multimodal approaches (including generic chemotherapy) with curative intent for cases detected by clinical examination~~ at health centres and first level hospital~~s	LMIC	Exact	**	750	Tufts GH-CEA registry
Treatment of early stage childhood cancers (such as Burkitt and Hodgkin lymphoma, acute lymphoblastic leukaemia, retinoblastoma and Wilms tumour) with curative intent in paediatric cancer units or hospitals	LMIC	Partial	**	1100	Tufts GH-CEA registry

Table S3: List of 13 interventions by cluster and platform which fulfilled all the 3 criteria of quality, technology, and comparator.

Interventions	Cluster	Platform	Quality	Technology	Comparator
Antenatal and postpartum education on birth spacing	RMNCH	Community level	✓	✓	✓
Childhood vaccination series (diphtheria, pertussis, tetanus, polio, BCG, measles, hepatitis B, HiB)	RMNCH	Community level	✓	✓	✓
Counselling of mothers on	RMNCH	Community	✓	✓	✓

providing thermal care for pre-term new-borns (delayed bath and skin to skin contact)		level			
Promotion of breastfeeding or complementary feeding by community health workers	RMNCH	Community level	✓	✓	✓
Early detection and treatment of NTDs	Communicable diseases	Community level	✓	✓	✓
Acute severe malnutrition management	RMNCH	Community level	✓	✓	✓
Detection and treatment of childhood infections with danger signs (IMCI)	RMNCH	Health Center	✓	✓	✓
Post-gender-based violence care, including counselling, provision of emergency contraception, and rape-response referral (medical and judicial)	RMNCH	Health Center	✓	✓	✓
Partner notification and expedited treatment for common STIs including HIV	Communicable diseases	Health Center	✓	✓	✓
Screening of HIV in all individuals with a diagnosis of active TB; if HIV infection is present, start (or refer for) ARV treatment and HIV care	Communicable diseases	Community level	✓	✓	✓

Screening for latent TB infection following a new diagnosis of HIV, followed by yearly screening among PLHIV at high risk of TB exposure; initiation of isoniazid preventive therapy among all individuals who screen positive but do not have evidence of active TB	Communicable diseases	Health Center	✓	✓	✓
Provision of aspirin for all cases of suspected acute myocardial infarction	NCD & IPC	Health Center	✓	✓	✓
Management of depression and anxiety disorders with psychological and generic antidepressants therapy	NCD & IPC	Health Center	✓	✓	✓
